# Facilitators and barriers for retention in HIV care between testing and treatment in Asia—A study in Bangladesh, Indonesia, Lao, Nepal, Pakistan, Philippines and Vietnam

**DOI:** 10.1371/journal.pone.0176914

**Published:** 2017-05-01

**Authors:** Sushil Koirala, Keshab Deuba, Oranuch Nampaisan, Gaetano Marrone, Anna Mia Ekström, for the CAT-S group

**Affiliations:** 1 Asia Pacific Network of People Living with HIV/AIDS, Bangkok, Thailand; 2 Department of Public Health Sciences, Karolinska Institutet, Stockholm, Sweden; 3 Department of Infectious Diseases, Huddinge, Karolinska University Hospital, Stockholm, Sweden; University of California, San Francisco, UNITED STATES

## Abstract

**Introduction:**

The need for efficient retention in HIV care is more evident than ever because of the expansion of earlier ART initiation and the shift towards ‘Test and Treat’. This study assesses factors affecting participation in the HIV care cascade among people living with HIV (PLHIV) in the Asia-Pacific Region.

**Methods:**

A total of 7843 PLHIV aged 18–50 years were recruited using targeted and venue-based sampling between October 1, 2012, and May 31, 2013, across 59 sites in 7 countries (Bangladesh, Indonesia, Lao People's Democratic Republic (Lao PDR), Nepal, Pakistan, Philippines and Vietnam). Statistically significant associations between demographic and health system determinants, and various steps in the HIV care cascade were computed using a generalized structural equation model.

**Results:**

A high proportion of PLHIV (40–51%) presented late for HIV care and delayed linkage to care in all seven countries. However, once PLHIV enrolled in care, retention in the various steps of the care cascade including adherence to antiretroviral treatment (ART) was satisfactory. The proportion still engaged in HIV care at 36 months post HIV diagnosis, varied from 78% in Nepal to >90% in Lao PDR. Similarly, the proportion of ART initiation who also were adherent to ART ranged from 91% in Bangladesh to >95% in Philippines/ Vietnam and from 70% in Lao PDR to 89% in the Philippines respectively. The following factors enhanced the likelihood of ART initiation and high adherence to HIV care and ART: good client-provider communication, high HIV treatment literacy, a referral from a health worker and TB/HIV co-infection. The following barriers were identified: young age, sex work, imprisonment, transgender identity, illiteracy, rural residence, alcohol/ injecting drug use, perceived poor health status, lack of health insurance, fear of confidentiality breach, self-referral for HIV testing, and public hospital as the place of HIV diagnosis.

**Conclusions:**

HIV programme planners should ensure easy access to HIV testing and earlier linkage to HIV care among PLHIV. In addition, multiple socio-economic and health systems barriers need to be addressed along the HIV care cascade to reach the UNAIDS 90-90-90 target in the Asia-Pacific region.

## Introduction

In the Asia-Pacific region, the overall number of new HIV infections declined by 31% between 2000 and 2014, reaching an estimated 340,000 new infections over this 15-year period. Some countries in the region have been particularly successful in reducing the incidence rate, such as Nepal, where new infections declined by 87%, and Thailand, where a 63% decline has been achieved since 2001[1]. However, other Asian countries have experienced opposite scenarios, such as Pakistan, with an 8-fold increase, Indonesia with a 2.6-fold increase, and the Philippines, where new infections have more than doubled since 2001[1]. The AIDS response in many Asian countries is heavily dependent on donor funding and therefore highly vulnerable given the size of the population at risk. The level of dependency varies from 58% of HIV care being financed by external sources in Indonesia to more than 90% in Nepal (84% in Bangladesh, 93% in Lao Peoples’ Democratic Republic (Lao PDR), 64% in Pakistan, 60% in Philippines and 68% in Vietnam) [2].

All 7 countries included in this study: Lao PDR, Bangladesh, Indonesia, Nepal, Pakistan, Philippines and Vietnam, are experiencing concentrated epidemics, defined as having an HIV prevalence below 1% in the general population but above 5% among key populations in local areas, such as men who have sex with men (MSM), transgender (TG), sex workers, and people who inject drugs. However, some local areas, such as the remote province of Papua in Indonesia, is experiencing a generalized epidemic (HIV prevalence >1% among pregnant women attending antenatal clinics)[3]. In fact, among certain Asian key populations, e.g., 53% of people who inject drugs in Faisalabad and 15% of transgender people in the Larkana areas of Pakistan, are infected with HIV. Likewise, HIV prevalence estimates are very high in Indonesia; 56% of people who inject drugs in Jakarta and 25% of female sex workers in the Jayawijaya area are living with HIV[2].

Antiretroviral therapy (ART) services have been rapidly scaled up in the Asia-Pacific region, in turn benefiting both survival and secondary prevention. The number of people who are living with HIV (PLHIV) who receive ART increased to >4-fold in last ten years, to a regional estimate of 1.8 million in 2014. Nevertheless, treatment coverage is still low. Around 50% of PLHIV in the region who are eligible for ART receive treatment overall [1, 4], but only 6–8% of PLHIV in Indonesia and Pakistan have access to ART. Consequently, AIDS-related mortality increased rapidly in these countries over the last decade (2005–2013), with a 4-fold increase in Indonesia and a 3.5 fold increase in Pakistan [5].

Recent findings from the strategic timing of antiretroviral treatment (START) study[6] conducted at 215 sites in 35 countries, revealed that starting ART immediately rather than later (CD4 counts <350 cells/μl or at AIDS-defining illness) resulted in a 53% reduction in risk of developing serious AIDS-related events (e.g., AIDS-related cancer) or non-AIDS related events (major cardiovascular, renal and liver disease, non-AIDS cancer) and death. Earlier HIV diagnosis and linkage to care also reduced all-cause mortality by 32% among PLHIV in a study from England and Wales [7]. In a population-based study from the United States, 20% of PLHIV delayed enrollment in HIV care for more than 3 months following their HIV diagnosis [7]. The majority of PLHIV in the Asia-Pacific region are not yet tested and aware of their HIV status [5], and therefore are not only facing unnecessary and preventable HIV/AIDS-related illness, but they are also at higher risk of infecting their partners [8, 9]. Those linked to care but with poor ART adherence may also develop viral drug resistance, ultimately affect both individual and population-wide treatment outcomes[10].

The need for efficient engagement in HIV care is more evident than ever because of the expansion of earlier ART initiation and the shift towards ‘Test and Treat’[11]. In 2014, UNAIDS proposed the 90-90-90 targets, i.e., that by 2020, 90% of PLHIV will know their HIV status, 90% of PLHIV who know their HIV status will receive HIV treatment, and 90% of PLHIV on ART should be virally suppressed [11, 12]. It is estimated that three million new HIV infections and three million AIDS-related deaths could be averted if these targets are achieved [11]. To reach these targets and to optimize treatment and prevention benefits of ART, early identification of all PLHIV, optimal linkage to and retention in HIV care, and sufficient use and optimal adherence to ART to achieve viral suppression are key[13, 14].

We sought to assess factors affecting retention in HIV care at the most crucial steps in the care cascade–HIV testing, linkage to HIV care, engagement in HIV care, initiation of ART, and ART adherence—for PLHIV in 7 countries in the Asia-Pacific region that each have concentrated epidemics but varying trends and health system approaches to implement HIV prevention and treatment. We also assessed the specific health system and behavioral obstacles that need to be overcome for at least some of these countries to reach the UNAIDS 90-90-90 target, given strong and sustained national commitment combined with strengthened donor support.

## Methods

### Study design and study population

We present baseline (cross-sectional) data from a prospective longitudinal study of community access to HIV treatment, care and support services (CAT-S) that involved 59 sites in 7 countries (Bangladesh, Indonesia, Lao PDR, Nepal, Pakistan, Philippines, Vietnam) in the Asia-Pacific region[15]. CAT-S is one of the key pillars of Monitoring Access to Treatment in Asia (MATA)—a tool to document, monitor, and advocate issues related to access to treatment, care and support for PLHIV over a longer term. CAT-S is funded by the Global Fund to Fight AIDS, Tuberculosis and Malaria (GFATM) Round 10 regional grant[15]. CAT-S recruited individuals (men, women and MSM/TG) who self-report being diagnosed with HIV infection at least three months before the date of interview. Participants were eligible for the study if they were 18 to 50 years of age and provided written informed consent to participate in the study.

### Setting and sample size

The study sites were selected to represent areas where the prevalence of HIV is known to be high and where different types of HIV care services are implemented. Between October 1, 2012, and May 31, 2013, 7843 study participants were recruited. The possible number of study participants from each site was decided based on consultation with national associations of PLHIV. Because stigma and discrimination against PLHIV are highly prevalent and many risk behaviors (injecting drug use and all or some aspects of sex work) are criminalized in several of the countries studied, sample size calculations could not be based on random recruitment of study participants. However, the involvement of national networks in each country helped us recruit a wide range of PLHIV (different age groups, sexual orientation and risk groups). This also helped us to conduct robust analyses about various issues of access to HIV treatment, care and support services.

### Study tools and sampling technique

An English version of a semi-structured questionnaire (S1 Table) was finalized by the technical team at Asia Pacific Network of People Living with HIV/AIDS (APN+) based in Bangkok, translated into different local languages (Bangla/Bengali, Lao, Indonesian, Urdu, Filipino, Nepali and Vietnamese), and pilot-tested in all countries between June and September 2012. A workshop was organized in each country to finalize the questionnaire (to check the wording of the questions and completeness of response categories). All the data collectors were PLHIV and priority was given to those with formal education, who were working as a peer/outreach educators, and who had some previous experience of data collection. In June 2012, a 4-day training session on field research implementation was organized by APN+ to inform data collectors about study implementation, the study objectives, procedures, informed consent process, interview techniques, and data coding and reporting.

Both targeted sampling and facility-based sampling were used to recruit study participants. A targeted sampling technique—which helped us understand the existence of different subgroups in a particular setting—was used to recruit study participants from identified geographical areas. Snowball sampling has been found effective for recruiting hidden populations, but increases the risk of oversampling easily available study participants and may also be too much influenced by the characteristics of the initial study recruits also called ‘seeds’. We, therefore, used modified targeted snowball sampling, assumed to be more effective and valid for recruiting hidden marginalized populations [16]. We call it modified snowball sampling because we followed two key steps before identifying ‘seeds’ that could facilitate the enrolment of study participants. In the first step, we identified areas within each of the seven countries where we could enroll study participants by using national and local networks of PLHIV. Most key populations are involved in smaller or larger networks of people who have similar behaviours and characteristics. Contact persons for the various networks also helped us to identify different key populations (sex workers, MSM and transgender and people who inject drugs living with HIV) in each study area. In the second step, we also performed a review of the secondary literature to estimate a possible number of individuals living with HIV in the country study areas. We thereafter selected ‘seeds’ in each of these study areas to enroll study participants.

Facility-based sampling techniques were used to recruit study participants from different institutions (health centers/hospitals, self-help groups, outreach/drop-in centers and different treatment, rehabilitation centers). Due to security issues in two Pakistani provinces—Khyber Pakhtoonkhwa bordering with Afghanistan (study sites-Peshawar and Swahi) and Baluchistan bordering with Iran and Afghanistan (Study sites- Quetta and Turbat)—help was sought from the local law-enforcing agencies and provincial AIDS control programs to recruit study participants. Data collection was carried out face-to- face (between October 2012 to May 2013) using the semi-structured piloted and translated questionnaire. The period includes a 3-month delay (between November 2012 and January 2013) due to typhoon risk in the Visayas and Mindanao areas of Philippines during this particular time period.

### Variables

The outcome variables were HIV testing, linkage to care, engagement in care, initiation of ART and ART adherence. We did not analyze the ultimate goal of HIV treatment (i.e. viral suppression) because more than 80% of our study participants from five studied countries never received a viral load test (Bangladesh 99.5%, Indonesia 81.9%, Nepal 87.8%, Philippines 92.0% and Vietnam 89.6%). HIV testing was assessed as a late presentation for HIV care (yes vs. no), which was defined as PLHIV presenting with CD4 cell count <200 cells/μl at the time of the first diagnosis of their HIV infection. Linkage to care was dichotomized as ‘timely meeting with health workers for HIV care’ (attending an appointment within 30 days of an HIV diagnosis) and timely CD4 testing (having had one or more documented CD4 tests within three months of an HIV diagnosis). Engaged in care was assessed as PLHIV who had at least one HIV medical care visit in the last 12 months. Initiation of ART was assessed as PLHIV who were eligible to take ART and also actually did receive ART. ART adherence over the past month was assessed by visual analog scale (VAS): “We would like to get your best guess about how much of your anti-HIV medication you have managed to take in the last month?” and asking the interviewee to estimate their adherence proportion (visual analog scale from 0% to 100% in 10% intervals; 0% meaning that the respondent has not taken any medication, and 100% meaning that s/he has taken every single dose) of their prescribed ART in the last month. ART adherence was later dichotomized into ≤90%: poor vs. >90%: good adherence. The VAS scale is a validated tool to measure the proportion of ART dosages taken over the last month[17]. Studies that validated VAS found that self-reported adherence to ART was consistent with electronic data and virological treatment outcome[17].

We analyzed different population and health-system level factors as independent variables (Table 1), following the theoretical framework of access to health care developed by Andersons[18] that influences the outcome variables.

**Table 1:** Independent variables.

Factors	Variables
**Population characteristics**	
**Predisposing factors**	
	**Demographics:** Age, risk groups and marital status.
	**Social structure:** Education, occupation, substance use (alcohol and injecting drug use), disclose HIV status to anyone except spouse, a close family member and doctor, discrimination, violence and housing instability.
	**Beliefs: **HIV treatment literacy (measured by 25 true/false items) and perceived confidentiality of the medical records relating to HIV status in the clinic/hospital PLHIV are visiting.
**Enabling factors**	
	Income, enrolled in any kind of health insurance program, cost to reach ART centre, distance to ART centre, duration of ART started, ART side effects and social support (Multidimensional Scale of Perceived Social Support).
**Need factors**	
	Perceived health status, reason for HIV testing, use of internet to find the HIV-related information, ever diagnosed with tuberculosis (TB) after becoming HIV positive (TB/HIV co-infection), and HIV/ hepatitis C virus (HCV) co-infection.
**Health system**	
**Resources/organization**	
	Received home-based care services, relationship with health workers (Patient Reactions Assessment), health care professional (for example, a doctor, nurse, counsellor, laboratory technician) ever told other people about HIV status without consent and place of HIV diagnosis (Government hospital while on treatment, Private hospital, HIV voluntary and counselling centre (VCT) in a hospital, VCT centre in non-governmental organization).

### Data management and analysis

An Electronic Data Capture system (iDataFax) was used to submit data to the Center of Excellence for Biomedical and Public Health Informatics (BIOPHICS) located at Mahidol University. Upon receipt of the data, data officer at the APN+ was responsible for a visual review of the data for missing values and query other inconsistencies in country specific data.

Statistically significant associations between independent variables and the outcome variables were computed using a generalized structural equation model (GSEM) using the ‘Bernoulli-Logit function’. GSEM is a more advanced and robust analysis than traditional analysis methods because it assesses the associations between multiple independent variables and outcome variables in the same model even if outcome variables are varied in nature (dichotomous, ordinal and count, etc.). GSEM also allowed us to address the complex sample survey design (7 countries and 59 study sites) in the analysis. Svy: gsem of STATA was used to fit the statistical model for complex survey data. Details about the GSEM model are provided below. The significance level was set at 0.05. STATA version 14.0 was used for all analyses.

The following covariates controlled in the final GSEM model. Additional covariates controlled for: Late presentation for HIV care- marital status; Linkage to care (health workers) model- age, risk groups (people who inject drugs, refugee or asylum seeker or internally displaced person), education, living area (urban, small town, and rural), alcohol drinking behavior and cost to reach ART center (median value <2 vs. > = 2 USD); Linkage to care (CD4 test) model- Age, risk groups (people who inject drugs, refugee or asylum seeker or internally displaced person), education, HIV treatment literacy, confidentiality of HIV related records in the clinics/hospital, income and TB-HIV co-infection; Engaged in care model- risk groups (transgender and female sex worker), marital status, HIV treatment literacy, income, perceived health status, social support, TB-HIV co-infection, and relationship with health workers (patient information index); ART initiation model- risk groups (transgender), education, disclose HIV status, housing instability, income, social support and ever used internet to find HIV related information; ART adherence model- Risk groups (men who have sex with men, people who inject drugs, and refugee or asylum seeker or internally displaced person and domestic migrant worker), education, ever used any illicit drugs by injection (current/past user and never), income, duration of ART started (<1 year, 1–3 years and >3 years), long term ART side effects (1–2 type of side effects/ more than 2 types of side effects/ none), perceived health status and relationship with health workers (patient reactions assessment).

### Ethical considerations

The study protocol was approved by the responsible research council of each country (Bangladesh: Bangladesh Medical Research Council; Indonesia: Atmajaya University; Lao PDR: National Ethics Committee for Health Research at the Department of Hygiene and Diseases Control, Ministry of Health; Nepal: Nepal Health Research Council; Pakistan: National Institution Bridge Consultants Foundation, Karachi; Philippines: Philippine Health Research Ethics Board; Vietnam: Ha Noi School of Public Health). A code of conduct to ensure confidentiality, questionnaire storage and analysis was developed and used. All the participants were adequately informed about the study objectives and the procedures at the beginning of the interview and assured about full anonymity and confidentiality. They were also made aware that participation was voluntary and that they were free to refuse to answer any question or to withdraw from the interview at any time. Those who were willing to give voluntarily written informed consent (thumbprint was obtained from those who were not able to sign) were recruited for the study. Participants were given monetary incentives (5 USD in Lao PDR, Indonesia, Nepal and Vietnam and 10 USD in Bangladesh, Pakistan and Philippines) for study participation.

## Results

**Fig 1:**
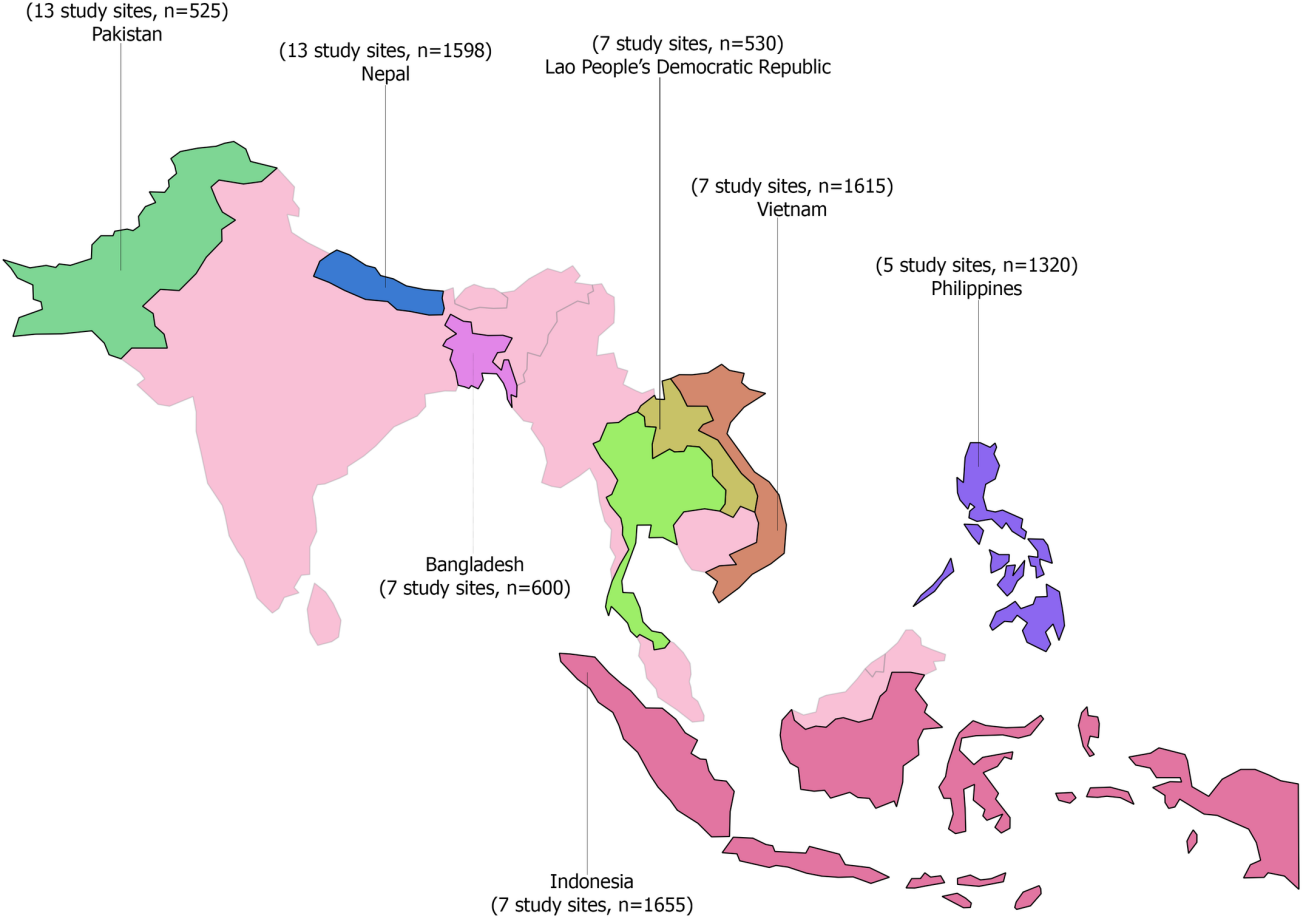
Study sites and sample sizes for seven countries in the Asia-Pacific region.

Fig 1 presents the study sites and the total sample sizes in each of the seven Asia-Pacific countries. In total, 7843 PLHIV were enrolled—with a mean age of 34 (6.9 SD) years of age—of which 80% identified themselves as male, 38% as female and 2.4% as transgender. The median duration on ART treatment was 3 years. Other characteristics of PLHIV are presented in Table 2.

**Table 2:** Characteristics of study participants (N = 7843).

**Population characteristics**	**Total (%)**
Age (years)	
Mean (SD)	33.9 (6.9)
**Gender**	
Male	4716 (80.1)
Female	2937 (37.5)
Transgender	190 (2.4)
Risk groups*	
Men who have sex with men/ Transgender	1651 (21.1%)
Lesbian	19 (0.2%)
Sex worker	824 (10.5%)
Injecting drug user	1,969 (25.2%)
Refugee or asylum seeker or Internally displaced person	270 (3.5%)
Domestic migrant worker	434 (5.6%)
International migrant worker	1,030 (13.2%)
Prisoner	202 (2.6%)
Others**	2,362 (30.2%)
**Marital Status**	
Married and currently living with partner	3,342 (42.6%)
Temporarily living with partner	247 (3.2%)
In a relationship, not living together	464 (5.9%)
Single	2,390 (30.5%)
Divorced/Separated	403 (5.1%)
Widow/Widower	994 (12.7%)
**Education**	
Illiterate	837 (10.7%)
Can read and write	714 (9.1%)
Primary completed	1,111 (14.2%)
Secondary completed	1,746 (22.3%)
Higher Secondary completed	2,024 (25.8%)
College level or high	1,361 (17.4%)
Others***	50 (0.6%)
**Occupation**	
Unemployed	1,792 (22.9%)
Employed	6,049 (77.1%)
**Living area**	
A rural area	2,167 (27.7%)
A small town	1,591 (20.3%)
A large town or city	4,077 (52.0%)
**Alcohol use**	
Current user	1,957 (25.0%)
Past user	1,535 (19.6%)
Never	4,351 (55.5%)
**Ever disclosed HIV status^a^**	
Yes	3,964 (50.5%)
No	3,879 (49.5%)
**Duration of ART started**	
Median (in years)	2.99
< 1 year	1,091 (18.4%)
1–3 years	2,198 (37.0%)
> 3 years	2,647 (44.6%)
**Short term ART side effects^b^**	
None	931 (15.7%)
1–2 type of side effects	1,327 (22.4%)
More than 2 types of side effects	3,668 (61.9%)
**Long term ART side effects^c^**	
None	1,322 (22.3%)
1–2 type of side effects	2,253 (38.0%)
More than 2 types of side effects	2,351 (39.7%)
**Denied health services because of your HIV status^d^**	
Yes	483 (7.8%)
**Verbally insulted, harassed and/or threatened^d^**	
Yes	1,304 (16.6%)
**Physically assaulted^d^**	
Yes	499 (6.4%)
**Housing instability because of HIV status^d^**	
Yes	469 (8.1%)
**HIV treatment literacy^e^**	
Median (range)	11 (0–23)
Poor	3491 (44.6%)
Good	4345 (55.4%)
**Perceived confidentiality of HIV records in clinic**	
Completely confidential	4,489 (62.4%)
Do not know	2,234 (31.0%)
Not kept confidential	475 (6.6%)
**Health worker ever disclose HIV status without consent**	
Yes	618 (8.6%)
No	4,519 (62.8%)
Not sure	2,060 (28.6%)

SD, Standard deviation; ART, Antiretroviral therapy. Note: percentage may not add up to 100 percent because of missing values and no response.

*Multiple responses allowed

**Do not belongs to any risk group, clients of sex worker, wives of migrants, partner of injecting drug users and wives or partner of PLHIV

***can sign only

^a^ Ever disclosed HIV status to anyone except your spouse, a close family member, and your doctor

^**b**^Nausea, vomiting, muscle aches and pains, diarrhea, fatigue, gas and bloating, hair loss, headache, sleeping difficulties and mood changes.

^c^diabetes, heart related diseases, body fat changes, numbness in the limbs, problem in kidney and liver, osteopenia/osteoporosis and jaundice

^d^In the last 12 months

^e^dichotomization based on sample median.

**Fig 2:**
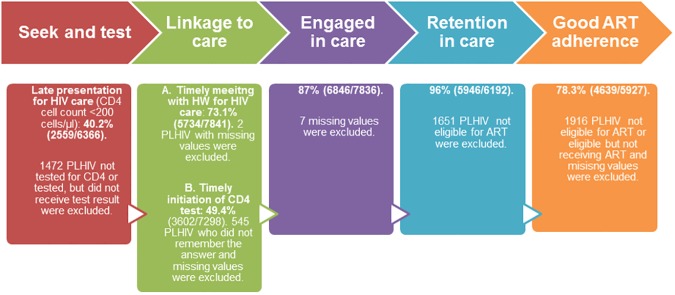
HIV testing, linkage to care, engaged in care, ART initiation and ART adherence among PLHIV in seven countries of the Asia-Pacific region.

As shown in Fig 2, four out of every 10 PLHIV presented late for HIV care. Of those diagnosed with HIV, 73% made an appointment with a health worker within 30 days of their diagnosis, but only 49% had a CD4 test done within 3 months of diagnosis. Among PLHIV, engagement in HIV care (87%; 6846/7836) and ART initiation (96%; 5946/6192) was high. Good ART adherence was reported by an average of 78% (4639/5927) across the seven countries. The outcomes of interest varied considerably in the seven countries studied especially late presentation for HIV care, timely appointment with a health worker for HIV care, having had a timely CD4 test, and ART adherence) but variation in the proportion of PLHIV engaged in HIV care and ART initiation was not significant (Table 3).

**Table 3:** HIV testing, linkage to care, engaged in care, ART initiation and ART adherence among PLHIV by country.

**Country**	**Bangladesh** **(%)**	**Lao PDR** **(%)**	**Nepal** **(%)**	**Pakistan** **(%)**	**Philippines** **(%)**	**Vietnam** **(%)**	**Indonesia** **(%)**
**Outcomes**							
HIV testing (late presentation for HIV care)	42.8	64.6	28.3	17.4	40.2	41.6	47
**Linkage to care**							
Timely meeting with health worker for HIV care	85.3	94.3	65.2	76.8	77.7	54.4	83
Timely CD4 test done	52.4	46.6	30.6	51.8	51.1	47.7	64.7
**Engaged in care**	93.2	99.8	77.9	86.1	89.2	92.2	84.6
**ART initiation**	90.6	99.8	95.1	93.5	97.9	97.7	95
**Poor ART adherence**	0.4	30.3	27.1	16.7	11.5	29.6	21.4

### HIV testing: PLHIV presenting with CD4 cell count <200 cells/μl at time HIV diagnosis

**Fig 3:**
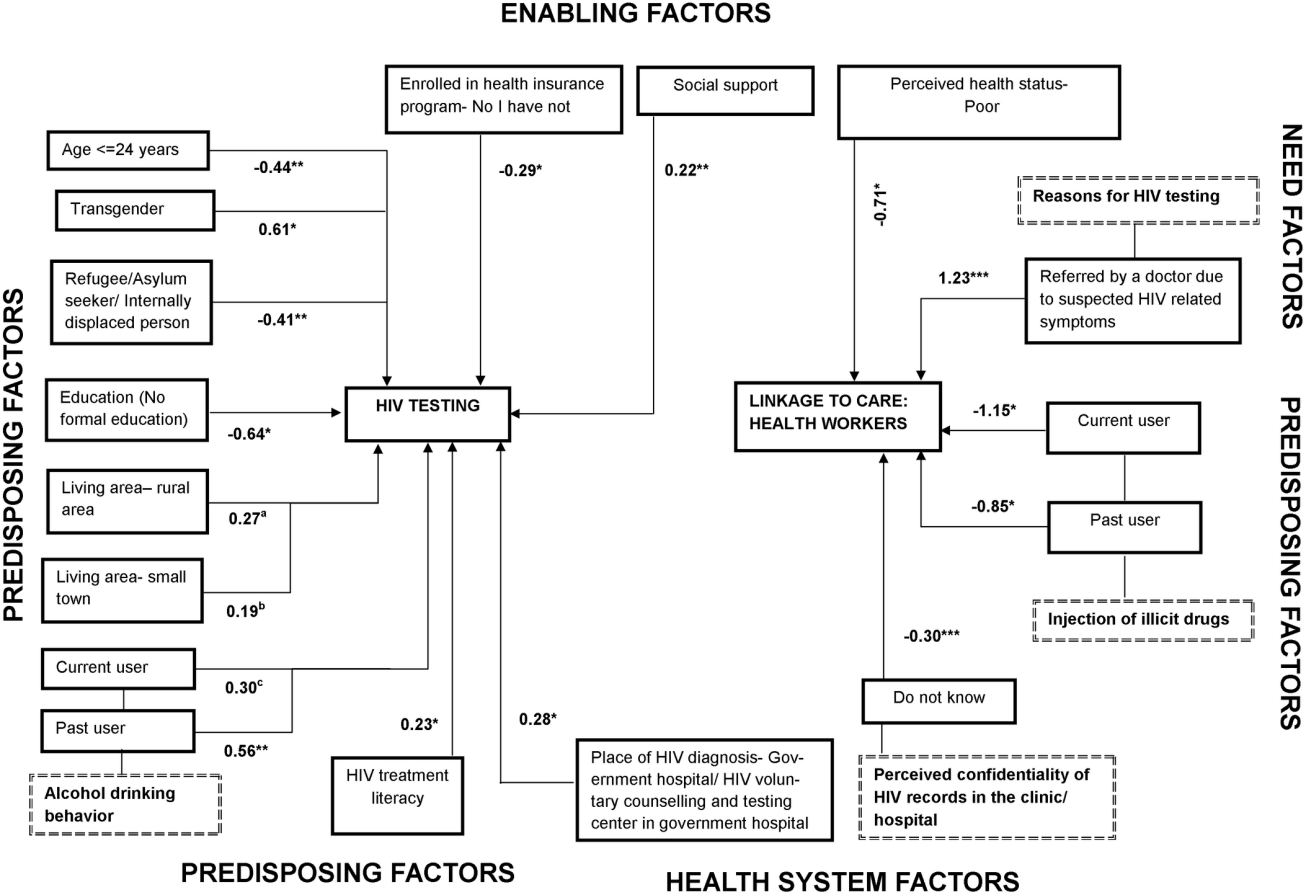
Factors associated with late presentation for HIV care and linkage to care (health workers) among people living with HIV in seven countries of the Asia-Pacific region. ¥ coefficients of factors of late presentation for HIV care and linkage to care based on generalized structural equation model (GSEM) * p ≤ .05; ** p < .01; *** p < .001; ^a^p = 0.083; ^b^ 0.073; ^c^p = 0.084.

As shown in Fig 3, those who identified as transgender and also were living with HIV had a significantly higher risk of presenting late for HIV care. Being a refugee/ asylum seeker/ or internally displaced and living with HIV was negatively associated with presenting late for HIV care. Young PLHIV (age ≤ 24 years) had a significantly lower risk of presenting late for HIV care. Different characteristics of PLHIV (past vs. current alcohol user, good vs. poor HIV treatment literacy, and place of HIV diagnosis (government hospital/ voluntary counselling center in public hospital vs. private hospital) was positively associated with presenting late for HIV care, whereas lacking formal education and health insurance were significantly and negatively associated. There was a marginally significant positive association between living area (rural/small town vs. urban) and late presentation for HIV care.

### Timely linkage to HIV care: Timely appointment with health workers for HIV care and/ or having a timely CD4 test performed

**Fig 4:**
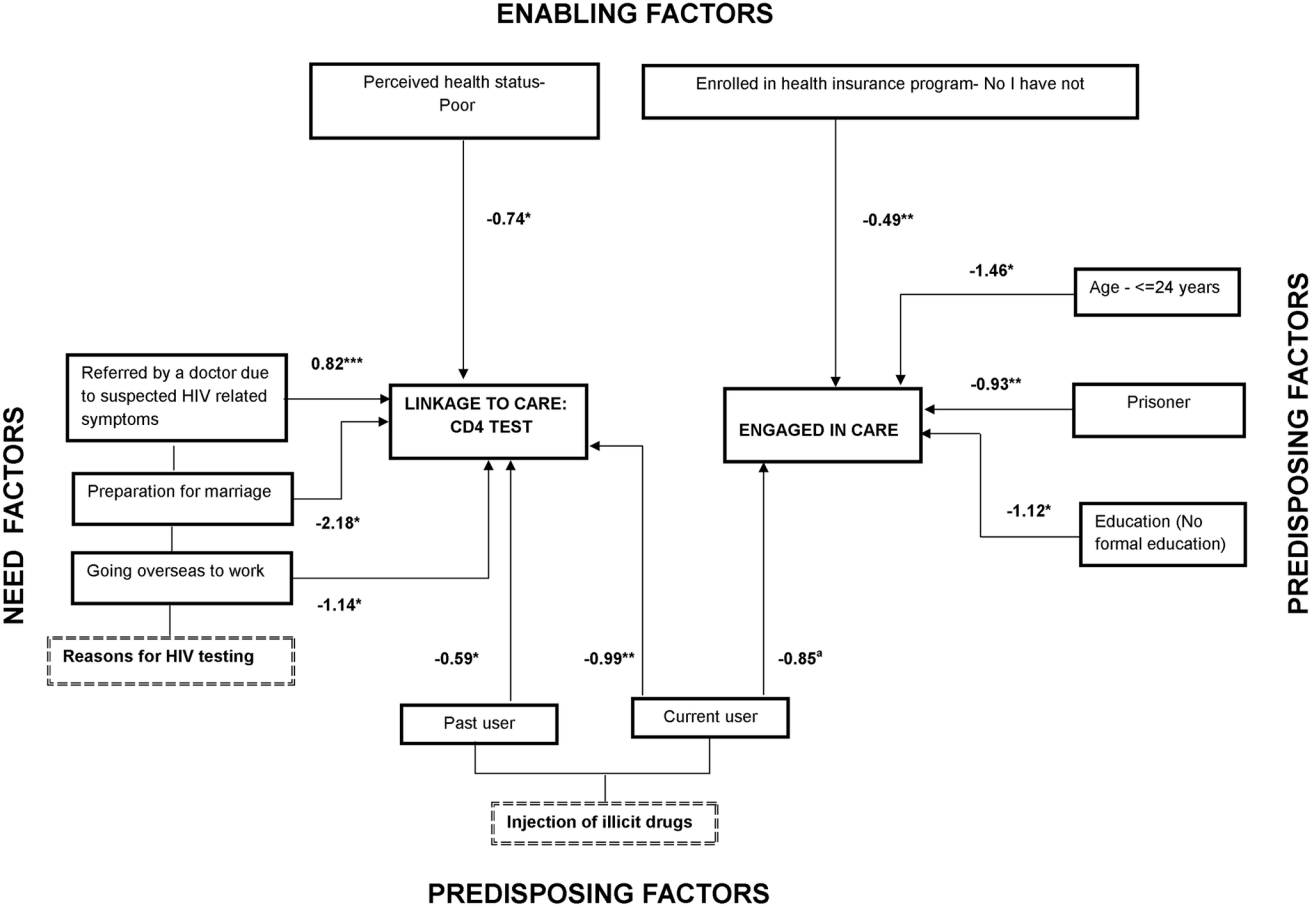
Factors associated with linkage to care (CD4 test) and engagement in care among people living with HIV in seven countries of the Asia-Pacific region. ¥ coefficients of factors of engagement in care, retention in care and poor ART adherence, based on generalized structural equation model (GSEM). * p ≤ .05; ** p < .01; ^a^p = .075.

As shown in Figs 3 and 4, being a current and/or past injecting drug user was significantly and negatively associated with timely linkage to HIV care. PLHIV who perceived their own health status as poor, and those who felt uncertain about whether or not their HIV-related medical records were kept confidential at the clinic/ hospital they were visiting, had a significantly lower chance of linking up to HIV care on time. In contrast, PLHIV who reported that a doctor had referred them for HIV testing were significantly more likely to meet with health workers for HIV care in a timely manner and to have a timely CD4 assessment. PLHIV who reported marriage preparations or that they were going overseas to work as a reason for taking an HIV test, as well as those who were injecting drugs users, had a significantly lower likelihood of receiving a timely CD4 assessment.

### Engaged in HIV care: PLHIV who had at least one HIV-related medical visit in the last 12 months

As shown in Fig 4, PLHIV ≤ 24 years of age, prisoners, those lacking formal education, and those who lacked health insurance, had a significantly lower chance of being engaged in HIV care. Current injecting drug use appeared to be negatively associated with engagement in HIV care, but was only marginally significant.

### ART initiation: PLHIV eligible for ART who also were started on ART

**Fig 5:**
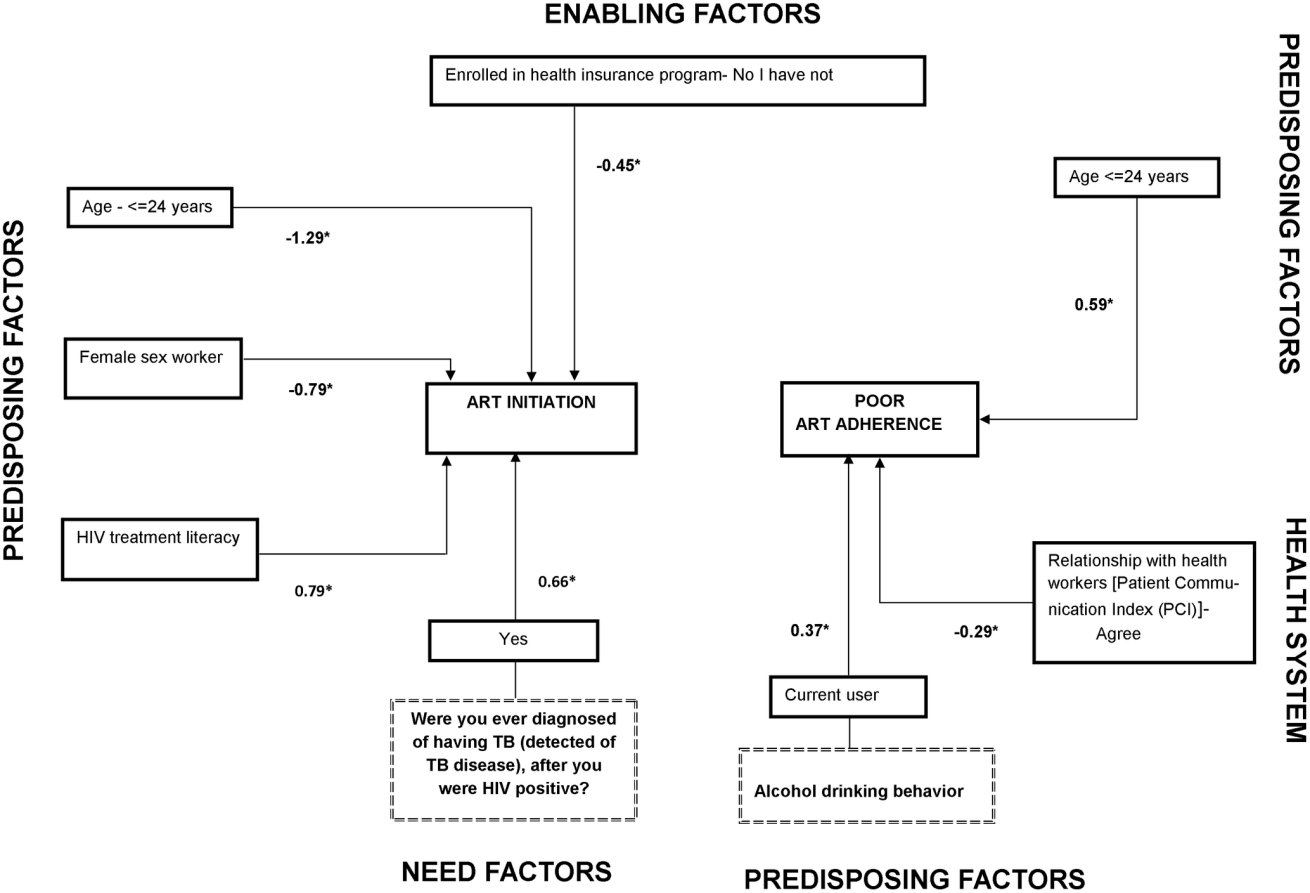
Factors associated with ART initiation and poor ART adherence among people living with HIV in seven countries of the Asia-Pacific region. ¥ coefficients of factors of engagement in care, retention in care and poor ART adherence, based on generalized structural equation model (GSEM). * p ≤ .05; ** p < .01.

As shown in Fig 5, PLHIV with good HIV treatment literacy, as well as those diagnosed with tuberculosis after HIV infection, had a significantly higher chance to initiate ART. On the other hand, PLHIV ≤ 24 years, female sex workers and PLHIV without health insurance all had a significantly lower chance to initiate ART.

### Poor ART adherence

At the national level, participants from Bangladesh reported the highest level of adherence to ART, while participants from the Lao PDR and Vietnam had the lowest level of adherence to ART (Table 3). Young people (≤ 24 years of age) and those reporting current alcohol use behavior had a significantly higher risk of poor ART adherence. PLHIV who were satisfied with the communication they had with their health workers about HIV infection and ART treatment had a good ART adherence (Fig 5).

## Discussion

We studied barriers and facilitators for uptake and retention in the HIV care cascade among 7843 PLHIV in seven countries in the Asia-Pacific Region. We found that lack of timely HIV diagnosis and poor linkage to care, including having a timely CD4 test assessment, are two major barriers to HIV care in this region. However, the proportions of PLHIV in other key steps of the HIV care continuum such as engagement in care and ART initiation (but not ART adherence) were above or close to the global 90-90-90 targets [11]. However, evidence suggests that progress in any single step of the HIV care cascade only has minimal impact on the overall goal, i.e., on increasing the high proportion of PLHIV with viral suppression [13]. Improved uptake and retention across all steps of the HIV care cascade are necessary; otherwise, multiple, serial barriers at each step of the cascade can hinder participation in subsequent steps[13]. Consistent with previous studies[19–27], we identified several population and health-system level barriers and facilitators of the HIV care continuum.

In our study, PLHIV who belonged to key populations (people who inject drugs, transgender, female sex workers, and prisoners), were less likely to be retained in HIV care, which is consistent with previous study findings [7, 19–22]. Different reasons have been reported for poor retention in HIV care among key populations, such as internalized stigma related to sex work and sexual orientation, discrimination, sex work environment, criminalization of risk behaviors (injecting drug use and sex work), and negative attitudes towards HIV treatment [20–22, 28, 29]. An American study found that transgender women living with HIV were less likely to be retained in HIV care because they prioritized hormone therapy and had concerns about adverse interactions between ART and hormone therapy [21]. Growing evidence suggests that the use of hormones without medical supervision is common among transgender in Asia [30] but its association with the HIV care continuum in those living with HIV is unexplored.

In the Asia-Pacific region, HIV prevalence rates vary between 10% in China and 20% in Vietnam among prisoners who inject drugs [31]. Sharing of contaminated injecting equipment and unprotected sex in prison settings makes prisoners a key population for HIV infection[28]. Poor quality HIV services and the transient nature of prisoners [32] often leads to poor ART access, adherence and retention in care. However, HIV management education in prison, as well as individual needs assessment and transportation assistance after release, are active interventions that could increase retention in HIV care among detainees[33].

Since 2005, the global scale-up of ART has contributed to a 35% reduction in overall HIV-related mortality. However, actions to reach young people have not been successful enough. AIDS mortality rates increased by 50% from 2005 to 2012 among young and adolescents worldwide [23]. Similar to previous research, our study also found that although young PLHIV (18–24 years of age) are more likely to ‘seek’ and ‘test’, they are less likely to engage in and retain HIV care [14, 19, 24, 34, 35]. ART adherence was also poorer among younger as compared to older PLHIV (aged ≥25). A recent review found individual and environmental barriers (stigma, discrimination, depression, lack of social support and lack of ‘youth-centered’ services) for adherence to and retention in care among young people and adolescents living with HIV [23].

Our findings suggest that PLHIV who were referred by a health worker for HIV testing due to suspected HIV-related symptoms were more likely to link to HIV care on time. This contrasts with previous findings from generalized epidemic suggesting that PLHIV who have self-referred for HIV testing are more likely to be linked to HIV care than are PLHIV referred by a health worker[36]. Our study also found that PLHIV who went for HIV testing in preparation for marriage or because they planned to work overseas, were less likely to be linked to HIV care. In consistent with findings of other study, those who test positive without actively wanting to be tested for HIV (and who do not have any HIV-related symptoms) are less likely to be linked to HIV care because they are less prepared for an HIV diagnosis, and less motivated [37]. In our study, self-reported poor health status was significantly and negatively associated with linkage to HIV care. PLHIV can avoid seeking care despite self-perceived poor health status because of multiple barriers and competing urgencies such as stigma, abuse from providers and risk of losing income[38].

Over the last five decades, the oil business and infrastructure development in the Gulf Cooperation Council countries has led to massive migration movements of unskilled workers (mainly male) from the Asia-Pacific region [39]. Migrant workers undergo mandatory HIV screening annually to renew their work permit [40] and get deported if they test HIV-positive without being linked to HIV care in most of these Gulf countries. Implementation of such discriminatory policies is a missed opportunity to catch and link migrant labourers to HIV care, but migrating for work is also an important barrier for linkage and retention in HIV care in the home countries [25].

Participants in our study who were diagnosed with HIV at government hospitals or public voluntary counseling and testing centers were more likely to present late for HIV care than those diagnosed at a private hospital. This supports findings from a multi-country analysis showing that individuals with higher household income were more likely to seek private sector HIV testing and care[41]. People with low socioeconomic status usually seek health service from the public sector because it costs significantly less than a private hospital. They are also less likely to seek health services and to present late for care because they cannot pay.

In our study, PLHIV not enrolled in any health insurance program were more likely to seek HIV testing but less likely to engage in HIV care and initiate ART. Enrollment in any health insurance program was lower in Indonesia (39%) and Vietnam (40%) than in the Philippines (85%) (data not shown). Insurance that covers health care among PLHIV does not exist in Nepal and Bangladesh. In Pakistan, PLHIV can enroll in a health insurance program, but detailed data is not available about coverage or impact on accessing health care among PLHIV.

None of our study participants from Lao PDR had enrolled in the available health insurance program. Four countries (Indonesia, Vietnam, Philippines and Lao PDR) provide antiretroviral drugs and CD4 cell count tests free of charge, and have social protection programs (*Asuransi Kesehatan untuk Keluarga Miskin*–*Askeskin* in Indonesia; Community-Based Health Insurance in Lao PDR; PhilHealth in Philippines; and Vietnamese Social Security Social Health Insurance Program). These programs prioritize health care and other services (monthly cash allowance for daily necessities and common drug provision) among PLHIV. However, studies conducted in Indonesia, Lao PDR and Vietnam found that PLHIV experiences a catastrophic financial burden while seeking HIV/AIDS-related health services which are not covered by social protection programs, such as viral load tests, hospital admission fees or diagnosis and treatment of comorbid diseases[3, 42, 43]. Serious loopholes also exist in available social protection programs, for example, Indonesian PLHIV who not yet are eligible for ART, need to pay for HIV care[3]. The coverage of social protection programs is also limited because of barriers such as lengthy administrative processes (to obtain an identification card and certificate of poverty), unclear procedures, and lack awareness about available social protection services among PLHIV[43–45].

Consistent with previous literature[26], our study found that PLHIV living in rural/small town were more likely to present late for HIV care than their urban counterparts. The most frequently reported barriers to HIV care were long distances to the health center, a shortage of health workers, a lack of access to transportation and community stigma toward PLHIV in the community [26].

PLHIV in our study who reported fear of confidentiality breach by health workers in relation to their HIV-related medical records were less likely to make timely appointments for HIV care. Nine percent of the PLHIV in our study reported that health workers disclosed their HIV status to others without their consent (widely differ between study countries). A study from Bangladesh found that 80% of the nurses and 90% of the physicians behaved discriminatory towards PLHIV[46]. Despite the adoption of guidelines to protect the human rights of PLHIV by different Asian countries, fear of a confidentiality breach is still frequently reported as a barrier for seeking HIV care [27, 47].

PLHIV with no formal education in our study were more likely to seek HIV testing and care, findings consistent with those in a previous study in which low literacy level was associated with acceptability of HIV testing in a health care setting[48]. Factors like trust and dependence on health workers may influence the association we observed between literacy level and acceptability of HIV testing. PLHIV with good HIV treatment literacy in our study were more likely to initiate HIV care than those with poor literacy. Knowledge of ART and what taking it would mean may encourage PLHIV to retain HIV care despite presenting late for it initially.

Prevalence of TB/HIV co-infection is slowly emerging in the Asia-Pacific region; estimated prevalence is 17.2%[49]. Five of the countries we studied (Bangladesh, Indonesia, Pakistan, Philippines and Vietnam) are on the list of 22 ‘high TB burden’ countries that accounted for 82% of all estimated cases in the world [49]. An encouraging finding of our study was that PLHIV who had co-infection with TB were more likely to initiate ART. Studies suggest that TB-related deaths among PLHIV have declined significantly in the Asia-Pacific region since 2004, in some countries, mortality rates have more than halved[49]. Despite this progress, access to HIV testing among TB patients in Asia ranges from 1–2% in Bangladesh and Indonesia to 100% in Bhutan and Brunei[49].

Consistent with previous studies [38, 50], we found that PLHIV who were satisfied with the information provided by their health worker were more likely to adhere to ART. Good communication between client and provider helps clients fully understand the CD4 level and the importance of adhering to ART, which ultimately foster adherences [38]. Alcohol consumption is associated with poor ART adherence and also negatively affects the survival of PLHIV through different pathways (e.g. alcohol-induced immunosuppression that exacerbates the HIV related immunosuppression which increased hepatotoxicity)[51]. A quarter of our study participants were current alcohol users, and this behavior was also associated with poor ART adherence in the past month.

In our study, 21% of PLHIV were not eligible for ART (based on CD4 cell count during this study period), and we did not analyze engagement in HIV care among PLHIV who were not eligible for ART. Retention in pre-ART care is poor in many settings. For example, only 49% of PLHIV in South Africa with an initial CD4 cell count above > 200 cells/μl returned for CD4 testing within the next 13 months [52]. Similarly, PLHIV not eligible for ART were 3.4-fold less likely to retain in HIV care than patients with lower CD4 counts in Western Kenya [52].

Some limitations of this study need to be considered. The cross-sectional nature of data limits our ability to estimate the number of PLHIV retained in HIV care over time after initiation of ART and its associated factors. The purposive sampling technique might overestimate some of the country-specific outcomes of interest (ART initiation) if PLHIV participated in our study differ from those PLHIV who were missed during enrollment or refused to participate in our study. For example, the outcome of interest (ART initiation) is overestimated if PLHIV who took part in our study were more aware of HIV care services because of their association with national HIV support networks than those PLHIV who were not in touch with a national network. Different studies found that the key populations (either HIV-positive or HIV-negative) experience a high burden of psychosocial health problems (depression, suicidality)[53–55] and growing evidence also suggest that suicidality associated with less likely to engage in HIV care [56]. Our study does not assess the burden of psychosocial health problems and its association with HIV care continuum.

In summary, our study to assess factors affecting participation in the HIV treatment cascade among PLHIV in the Asia-Pacific Region showed a high proportion of PLHIV presented late for HIV care and delayed linkage to care. Despite this, their participation in the other steps of the HIV care cascade (engaged in and retention in HIV care/ART adherence) was encouraging. A large study (named SEARCH) implemented in rural Kenya and Uganda has already exceeded UNAIDS targets of 90-90-90 (for example estimate is above 90%) [57]. SEARCH study provided a comprehensive package (appointment reminders, quarterly visits with patient-centered care providing reduced waiting and overall visit duration) to increase demand for testing, improve treatment initiation, linkage to care and retention in HIV care. The effectiveness of similar interventions needs to be evaluated in the Asia-Pacific region. We also identified several population (young PLHIV, HIV treatment literacy, health insurance) and health system (confidential handling of HIV-related records) facilitators and barriers that need to be addressed to improve outcomes along the HIV care cascade in the study countries.

## Supporting information

S1 Table Study questionnaire. (PDF)
