# Supplementary material for: Facilitators and barriers for retention in HIV care between testing and treatment in Asia—A study in Bangladesh, Indonesia, Lao, Nepal, Pakistan, Philippines and Vietnam
Source: PLoS One. 2017 May 1;12(5):e0176914. doi: 10.1371/journal.pone.0176914 (PMC5411091; doi:10.1371/journal.pone.0176914)
Supplement: S1 Table — (PDF) [file pone.0176914.s001.pdf]

CATS : Community Access to Treatment, Care and Support Study
National PLHIV Networks and Asia Pacific Network of People Living with HIV/AIDS (APN+)

INTERVIEW QUESTIONNAIRE

A. Identification Tags

A1. Respondent ID

Country Code	Site Code	Data Collector Code	Respondent Serial No.
☐☐	☐☐	☐☐	☐☐☐☐

A2. Place of enrollment

☐ 1 Community ☐ 2 Health services centre ☐ 3 Hospital

☐ 4 Self-help group ☐ 5 Others, specify ______

A3. Name of the site (Facility) ______

A4. Location (Name of the City/Town/Village) ______

A5. Interviewer's name ______

A6. Interview start time ☐☐.☐☐ (*hh* . *mm*)

A7. Date of interview ☐☐ ☐☐ 2 0 1 ☐ (*dd* *mm* *yyyy*)

Before the Interview

☐ Inclusion criteria verified

1. Ask if the respondent is aged between 18-50 years.
2. Ask if the respondent is diagnosed of HIV at least three months prior to the date of interview.

☐ Informed content obtained

1. Please give information sheet to the respondent and allow him/her to read it. Please read the information for the respondent if s/he is not able to read it.
2. Please read the informed consent form for the respondent. If the respondent agrees to participate in the study "voluntarily", obtain his/her signature or thumb print (if the respondent is unable to write) in the form

CATS : Community Access to Treatment, Care and Support Study
National PLHIV Networks and Asia Pacific Network of People Living with HIV/AIDS (APN+)

SECTION 1 — BACKGROUND INFORMATION SHEET 1

Please tick the correct answer or write clearly in the space provided

1-1. How old are you? ☐☐ *years*

1-2. Sex

☐1 Male ☐2 Female ☐3 Transgender

1-3.What is your current relationship status?

☐1 Married or cohabiting and husband/wife/partner is currentry living in the same household

☐2 Married or cohabiting but husband/wife/partner is temporarily living/working away from the household

☐3 In a relationship but not living together

☐4 Single ☐5 Divorced/separated ☐6 Widow/widower

1-4. What is your level of education?

☐1 Illiterate ☐2 Can read & write ☐3 Primary completed

☐4 Secondary completed ☐5 Higher secondary completed ☐6 College level or higher

☐7 Others, specify ________

1-5. What is your present job?

☐1 Office employee ☐2 Self-employed ☐3 Work in another person's shop

☐4 Unemployed ☐5 Others, specify ________

1-6. Are you a member of NGO or National Networks or any CBO?

☐1 Yes, specify ________ ☐2 No

1-7. Do you have means to earn money/income? ☐1 Yes ☐2 No ***(Skip to Ques. 1-9)***

1-8. In average, how much do you earn every month?

Net amount in local currency ☐☐☐,☐☐☐,☐☐☐

1-9. How many people currently live in your household (including yourself), please share how old are they?

1-9.1 Total ☐☐ 1-9.2 Children (0-14 yrs) ☐☐

1-9.3 Youth (15-24 yrs) ☐☐ 1-9.4 Adults (above 24 yrs) ☐☐

1-10. Do you have any children in your household whose either one or both of the parents died of AIDS? If yes, how many?

☐1 Yes, number of orphans ☐☐ ☐2 No ☐3 Don't know/Not sure

1-11. How do you describe area that you live in?

☐1 A rural area ☐2 A small town ☐3 A large town or city

CATS : Community Access to Treatment, Care and Support Study
National PLHIV Networks and Asia Pacific Network of People Living with HIV/AIDS (APN+)

SECTION 1 — BACKGROUND INFORMATION SHEET 2

1-12. If you think of past one year, what was the average monthly income of your family (family means number of people including yourself who are living in the same house and share living costs)?

Amount in local currency ☐☐☐,☐☐☐,☐☐☐

1-13. Do you belong to, or have you in the past belonged to, any of the following categories?
(Tick at least one box. You can tick more than one if appropriate) (Interviewer: You do not need to read the answers)

- ☐ 1 Men who have sex with men
- ☐ 2 Lesbian
- ☐ 3 Transgender
- ☐ 4 Sex worker
- ☐ 5 Injecting drug user
- ☐ 6 Refugee or asylum seeker
- ☐ 7 Internally displaced person
- ☐ 8 Domestic migrant worker
- ☐ 9 International migrant worker
- ☐ 10 Prisoner
- ☐ 11 Others, specify ____________

CATS : Community Access to Treatment, Care and Support Study
National PLHIV Networks and Asia Pacific Network of People Living with HIV/AIDS (APN+)

SECTION 2 — HIV DIAGNOSIS AND HEALTH CARE SHEET 1

2-1. When were you diagnosed with HIV?

☐1 It was ☐☐ *years* ☐☐ *months* ago ☐2 I can't/don't remember

2-2. Why were you tested for HIV? *(Multiple choices question)*

☐1 I was going overseas to work
☐2 I was pregnant
☐3 To prepare for marriage
☐4 I was referred by a doctor due to suspected HIV-related symptoms or being sick (e.g. TB)
☐5 Husband/wife/partner/child tested positive
☐6 Illness or the death of husband/wife/partner/child
☐7 I just wanted to know the result
☐8 I had risky behaviour
☐9 I never choose to be tested, I was told I am HIV-positive by doctor/nurse
☐10 I was tested because of my work requirement in the overseas
☐11 During blood transfusion
☐12 Others, specify ____________

2-3. Where did you have your HIV diagnosed?

☐1 Government hospital while on treatment
☐2 Private hospital
☐3 VCT centre in a hospital
☐4 VCT centre in NGO
☐5 Others, specify ____________

2-4. How long did it take you to get your first CD4 count, after your HIV diagnosis (HIV positive result)?
(Please ask the approximate date if the respondent cannot remember the exact date)

☐1 Yes, it was ☐☐ *years* ☐☐ *months* ☐☐ *days* after the HIV diagnosis
(Please enter 0 day if the CD4 count was checked on the same day)

Also specify the CD4 count ☐☐☐☐

☐2 I have not had a CD4 count yet

☐3 I cannot remember *(Please prompt the answer first. If the respondent cannot remember the answer, tick here)*

2-5. Did you get your viral load test/examination after your HIV diagnosis? If yes, how long did it take you to get your first viral load test/examination after your HIV diagnosis? *(Please ask the approximate date if the respondent cannot remember the exact date)*

☐1 Yes, it was ☐☐ *years* ☐☐ *months* ☐☐ *days* after the HIV diagnosis
(Please enter 0 day if the viral load test was checked on the same day)

Also specify the viral load count ☐☐☐☐

☐2 I have not had a viral load test yet

☐3 I cannot remember *(Please prompt the answer first. If the respondent cannot remember the answer, tick here)*

CATS : Community Access to Treatment, Care and Support Study
National PLHIV Networks and Asia Pacific Network of People Living with HIV/AIDS (APN+)

SECTION 2 — HIV DIAGNOSIS AND HEALTH CARE SHEET 2

2-6. After HIV diagnosis, how long did it take you to meet the doctor/nurse/health worker?

- ☐1 Right after diagnosis, same day
- ☐2 It was ☐☐ *years* ☐☐ *months* ☐☐ *days* after HIV diagnosis
- ☐3 Not visited yet ***(Skip to Ques.2-9)***

2-7. Where did you go to meet the doctor/nurse/health worker?

- ☐1 Government hospital/clinic
- ☐2 Private hospital/clinic
- ☐3 NGO clinic
- ☐4 Others, specify ____________

2-8. After being HIV positive, how frequently do you visit your doctor/nurse/health worker?

- ☐1 Once a week
- ☐2 Once in a month
- ☐3 Once in every 2-3 months
- ☐4 Once in every 4-6 months
- ☐5 Only in every 7-12 months
- ☐6 Only when I am sick

2-9. Have you ever worked in HIV/AIDS program?

- ☐1 Yes for how long ☐☐ *years* ☐☐ *months* and please specify position ____________
- ☐2 No

2-10.Are you enrolled in any kind of health insurance program?

- ☐1 Yes
- ☐2 No, we don't have such scheme in our area
- ☐3 No, I have not

2-11.In the past 3 months, did you receive a home-based care services (or did somebody from NGO/hospital/ clinic visited your home and talked to you about your health, medicine, or your problems)?

- ☐1 Yes
- ☐2 No

CATS : Community Access to Treatment, Care and Support Study
National PLHIV Networks and Asia Pacific Network of People Living with HIV/AIDS (APN+)

SECTION 3 — HEALTH SEEKING BEHAVIOUR / ACCESS TO GENERAL HEALTH CARE AND ASSOCIATE COST SHEET 1

The following section is designed to understand the major disease that the respondents suffered from in past 6 months. There are three sections to record the three main diseases. This section is designed to understand the burden due to opportunistic infections. Please be mindful not to record information on minor illness.

3-1. In the past 6 months, did you suffer from any disease/health problem?

☐1 Yes, ☐☐ *times*; please provide the detail information of your illness below ☐2 No ***(Skip to Section 4)***

3-2. What was the 1st problem?

(a) Problem ________

(b) Did you take any medication by yourself? ☐1 Yes ☐2 No

(c) Whom did you consult with? Who was 1st, 2nd & 3rd contact person?

- 1st contact ________
- 2nd contact ________
- 3rd contact ________
- No, provide reason ________

(d) What was the diagnosis? ________

(e) Did you require hospitalization? ☐1 Yes, for how long ☐☐ *days* ☐2 No

(f) How much did you spend from your pocket for each of the following categories (amount in local currency)?

- Medicines ☐☐☐,☐☐☐,☐☐☐
- Consultation fee ☐☐☐,☐☐☐,☐☐☐
- Laboratory fee & diagnostics ☐☐☐,☐☐☐,☐☐☐
- Procedures or hospitalization ☐☐☐,☐☐☐,☐☐☐
- Transportation, foods & accommodation ☐☐☐,☐☐☐,☐☐☐
- Total cost ☐☐☐,☐☐☐,☐☐☐

(g) How did you pay for it? *(Multiple responses if possible)*

☐1 I paid from my pocket ☐2 My family paid for it

☐3 I got subsidy from other sources, specify ________

☐4 Others, specify ________

CATS : Community Access to Treatment, Care and Support Study
National PLHIV Networks and Asia Pacific Network of People Living with HIV/AIDS (APN+)

SECTION 3 — HEALTH SEEKING BEHAVIOUR / ACCESS TO GENERAL HEALTH CARE AND ASSOCIATE COST SHEET 2

3-3. If the respondent had more than one disease, Please ask what was the 2nd problem? *(if the respondent had only one problem, **Skip to Section 4)***

(a) Problem ______

(b) Did you take any medication by yourself? ☐1 Yes ☐2 No

(c) Whom did you consult with? Who was 1st, 2nd & 3rd contact person?

- 1st contact ______
- 2nd contact ______
- 3rd contact ______
- No, provide reason ______

(d) What was the diagnosis? ______

(e) Did you require hospitalization? ☐1 Yes, for how long ☐☐ *days* ☐2 No

(f) How much did you spend from your pocket for each of the following categories (amount in local currency)?

- Medicines ☐☐☐,☐☐☐,☐☐☐
- Consultation fee ☐☐☐,☐☐☐,☐☐☐
- Laboratory fee & diagnostics ☐☐☐,☐☐☐,☐☐☐
- Procedures or hospitalization ☐☐☐,☐☐☐,☐☐☐
- Transportation, foods & accommodation ☐☐☐,☐☐☐,☐☐☐
- Total cost ☐☐☐,☐☐☐,☐☐☐

(g) How did you pay for it? *(Multiple responses if possible)*

☐1 I paid from my pocket ☐2 My family paid for it

☐3 I got subsidy from other sources, specify ______

☐4 Others, specify ______

CATS : **Community Access to Treatment, Care and Support Study**
National PLHIV Networks and Asia Pacific Network of People Living with HIV/AIDS (APN+)

SECTION 3

HEALTH SEEKING BEHAVIOUR / ACCESS TO GENERAL HEALTH CARE AND ASSOCIATE COST SHEET 3

3-4. If the respondent had more than two diseases, Please ask what was the 3rd problem? *(if the respondent had only two problems, **Skip to Section 4)***

(a) Problem ______

(b) Did you take any medication by yourself? ☐1 Yes ☐2 No

(c) Whom did you consult with? Who was 1st, 2nd & 3rd contact person?

- 1st contact ______
- 2nd contact ______
- 3rd contact ______
- No, provide reason ______

(d) What was the diagnosis? ______

(e) Did you require hospitalization? ☐1 Yes, for how long ☐☐ *days* ☐2 No

(f) How much did you spend from your pocket for each of the following categories (amount in local currency)?

- Medicines ☐☐☐,☐☐☐,☐☐☐
- Consultation fee ☐☐☐,☐☐☐,☐☐☐
- Laboratory fee & diagnostics ☐☐☐,☐☐☐,☐☐☐
- Procedures or hospitalization ☐☐☐,☐☐☐,☐☐☐
- Transportation, foods & accommodation ☐☐☐,☐☐☐,☐☐☐
- Total cost ☐☐☐,☐☐☐,☐☐☐

(g) How did you pay for it? *(Multiple responses if possible)*

☐1 I paid from my pocket ☐2 My family paid for it

☐3 I got subsidy from other sources, specify ______

☐4 Others, specify ______

CATS : **Community Access to Treatment, Care and Support Study**
National PLHIV Networks and Asia Pacific Network of People Living with HIV/AIDS (APN+)

SECTION 4 — ACCESS TO SOCIAL SUPPORT

This section is designed to understand feeling of the respondent on each of the statements. Please explain to the respondent that this section is designed to understand his/her feelings on the kind of social support that they are getting. The respondent has to rate the answers within 5 choices. Please read the 5 choices loudly. Request the respondent to listen to the statements and think carefully and tell on how strongly they agree or disagree with the statement

	**Strongly disagree (1)**	**Disagree (2)**	**Neutral (3)**	**Agree (4)**	**Strongly agree (5)**
4-1. There is a special person who is around when I am need.	☐1	☐2	☐3	☐4	☐5
4-2. There is a special person with whom I can share my joys and sorrows.	☐1	☐2	☐3	☐4	☐5
4-3. My family really tries to help me.	☐1	☐2	☐3	☐4	☐5
4-4. I get the emotional help and support I need from my family.	☐1	☐2	☐3	☐4	☐5
4-5. I have a special person who is a real source of comfort to me.	☐1	☐2	☐3	☐4	☐5
4-6. My friends really try to help me.	☐1	☐2	☐3	☐4	☐5
4-7. I can count on my friends when things go wrong.	☐1	☐2	☐3	☐4	☐5
4-8. I can talk about my problems with my family.	☐1	☐2	☐3	☐4	☐5
4-9. I have friends with whom I can share my joys and sorrows.	☐1	☐2	☐3	☐4	☐5
4-10.There is a special person in my life who cares about my feelings.	☐1	☐2	☐3	☐4	☐5
4-11.My family is willing to help me make decisions.	☐1	☐2	☐3	☐4	☐5
4-12.I can talk about my problems with my friends.	☐1	☐2	☐3	☐4	☐5

CATS :	Community Access to Treatment, Care and Support Study<br>National PLHIV Networks and Asia Pacific Network of People Living with HIV/AIDS (APN+)
**SECTION 5**	**STIGMA & DISCRIMINATION**

5-1. Have you ever disclosed your HIV status to anyone except your spouse, a close family member, and your doctor?

☐1 Yes ☐2 No

5-2. In the last 12 months, how often have you been excluded from social gatherings or activities?

(e.g. weddings, funerals, parties, clubs)

☐1 Never ☐2 Once ☐3 A few times ☐4 Often

5-3. In the last 12 months, how often have you been verbally insulted, harassed and/or threatened?

☐1 Never ☐2 Once ☐3 A few times ☐4 Often

5-4. In the last 12 months, how often have you been physically assaulted?

☐1 Never ***(Skip to Ques.5-6)*** ☐2 Once ☐3 A few times ☐4 Often

5-5. If so, who physically assaulted you?

☐1 My husband/wife/partner ☐2 Another member of the household

☐3 Person(s) outside the household who is/are known to me ☐4 Unknown person(s)

5-6. In the last 12 months, how often have you been forced to change your place of residence or been unable to rent accommodation because of your HIV status?

☐1 Never ☐2 Once ☐3 A few times ☐4 Often

☐5 I have not disclosed my HIV status to public

5-7. In the last 12 months, how often has your child/children been dismissed, suspended or prevented from attending an educational institution because of your HIV status?

☐1 Never ☐2 Once ☐3 A few times ☐4 Often

☐5 Not applicable (I don't have children) ☐6 I have not disclosed my HIV status to public

5-8. In the last 12 months, how often have you been denied health services because of your HIV status?

☐1 Never ☐2 Once ☐3 A few times ☐4 Often

☐5 Not applicable (I was not sick) ☐6 I have not disclosed my HIV status to the health care provider ***(Skip to Section 6)***

5-9. Has a health care professional (for example, a doctor, nurse, counsellor, laboratory technician) ever told other people about your HIV status without your consent?

☐1 Yes ☐2 No ☐3 Not sure

5-10. How confidential do you think the medical records relating to your HIV status are in the clinic/hospital you are visiting?

☐1 I am sure that my medical records will be kept completely confidential

☐2 I don't know if my medical records are confidential

☐3 It is clear to me that my medical records are not being kept confidential

CATS : **Community Access to Treatment, Care and Support Study**
National PLHIV Networks and Asia Pacific Network of People Living with HIV/AIDS (APN+)

SECTION 6 — GENERAL HEALTH AND HIV RISK BEHAVIOURS SHEET 1

This section is designed to understand the issues related to general health and HIV risk behaviours among respondents. Please inform the respondent that you are also going to ask some questions related to drug, alcohol use and sexual behaviour. Inform that you are aware that it is not easy for respondents to answer some of the personal question. Inform the respondent that those information are necessary to understand how best programs should address the issues on health and HIV. Re-assure that an honest answer will help us greatly in understanding the real situation. Re-emphasize that all the answers will be kept confidential

6-1. In general, how would you describe your health at the moment?

☐1 Excellent ☐2 Very Good ☐3 Good

☐4 Fair ☐5 Poor

6-2. Think about past one month in general and tell us how many hours are you spending everyday (24 hours) doing the following activities? (Please note that the total has to be 24 hours)

6-2.1 Sleeping/sitting idle ☐☐ *hrs*

6-2.2 Walking/running or any other form of excercise ☐☐ *hrs*

6-2.3 Work that does not require hard labor (desk job, working in the kitchen etc.) ☐☐ *hrs*

6-2.4 Work that needs hard labor (agriculture, construction etc) ☐☐ *hrs*

6-3. Have you ever used any illicit drugs?

☐1 Yes, currently for ☐☐ *years* ☐☐ *months*

☐2 Yes, in the past about ☐☐ *years* ☐☐ *months ago*

☐3 No ***(Skip to Ques.6-7)***

6-4. Have you ever used any illicit drugs by injection?

☐1 Yes, currently for ☐☐ *years* ☐☐ *months*

☐2 Yes, in the past about ☐☐ *years* ☐☐ *months ago* ***(Skip to Ques.6-6)***

☐3 No ***(Skip to Ques.6-7)***

6-5. In last 6 months, did you receive adequate supply of clean syringes?

☐1 Every time I needed ☐2 Most of the time ☐3 Only sometimes ☐4 Never

6-6. Are you ever enrolled into Opioid Substitution Therapy (OST) (MMT/Oral Buprenorphine Therapy program)?

☐1 Yes, ☐☐ *years* ☐☐ *months ago* ☐2 No

CATS : Community Access to Treatment, Care and Support Study
National PLHIV Networks and Asia Pacific Network of People Living with HIV/AIDS (APN+)

SECTION 6 — GENERAL HEALTH AND HIV RISK BEHAVIOURS SHEET 2

6-7. Do you drink alcohol?

☐1 Yes, currently for ☐☐ *years* ☐☐ *months*

☐2 Yes, in the past about ☐☐ *years* ☐☐ *months ago* , for ☐☐ *years* ☐☐ *months* ***(Skip to Ques.6-9)***

☐3 No ***(Skip to Ques.6-9)***

6-8. How often do you drink alcohol in a week?

☐1 Daily ☐2 Nearly every day ☐3 3 or 4 times a week

☐4 Once or twice a week ☐5 Only sometimes (once or twice a month)

6-9. Do you smoke?

☐1 Yes, currently for ☐☐ *years* ☐☐ *months*

☐2 Yes, in the past about ☐☐ *years* ☐☐ *months ago* , for ☐☐ *years* ☐☐ *months* ***(Skip to Ques.6-14)***

☐3 No ***(Skip to Ques.6-14)***

6-10.Let's think about a month till today, and please tell me on average, how many cigarettes did you smoke each day? ☐☐ *sticks / day*

6-11.Have you cut down or increased smoking since you found out you were HIV positive, or do you smoke about the same amount?

☐1 Cut down ☐2 Increased ☐3 About the same amount ☐4 Stopped

6-12.Here are some different thoughts that smokers may have about quitting smoking. Please interact with respondents on their intentions to quit smoking and identify how best the following statements represent how respondents feel.

☐1 I have quit smoking & I will never smoke again	☐5 I definitely plan to quit smoking in the next 6 months	☐9 I never think about quitting smoking, and I have no plans to quit
☐2 I have quit smoking, but I still worry about slipping back, so I need to keep working on living smoke free	☐6 I often think about quitting smoking, but I have no plans to quit	☐10 I enjoy smoking & have decided not to quit smoking for my lifetime. I have no interest in quitting
☐3 I still smoke, but I have begun to change, like cutting back on the numberof cigarettes I smoke. I am ready to set a quit date.	☐7 I sometimes think about quitting smoking, but I have no plans to quit	
☐4 I definitely plan to quit smoking within the next 30 days	☐8 I rarely think about quitting smoking and I have no plans to quit	

6-13.During any visit to a hospital or clinic in the past 12 months, were you asked if you smoke tobacco by your health care provider (doctor/nurse/health worker)?

☐1 Yes ☐2 No

CATS : Community Access to Treatment, Care and Support Study
National PLHIV Networks and Asia Pacific Network of People Living with HIV/AIDS (APN+)

SECTION 6 — GENERAL HEALTH AND HIV RISK BEHAVIOURS SHEET 3

6-14.Do you have a spouse/steady partner? (Steady partner is for this study partner in relationship for at least 3 months)

☐1 Yes ☐2 No ***(Skip to Ques.6-20)***

6-15.What is the HIV status of your spouse/partner?

☐1 Positive ☐2 Negative ***(Skip to Ques.6-17)***

☐3 Don't know/Not known ***(Skip to Ques.6-17)***

6-16.If HIV positive, is s/he taking ART?

☐1 Yes ☐2 No

☐3 Don't know/Not known

6-17.In the past 6 months, have you had sex with your spouse/steady partner?

☐1 Yes ☐2 No ***(Skip to Ques.6-20)***

6-18.In the past 6 months, how frequently did you use condoms when you had sex with him/her?

☐1 Never ☐2 Sometimes

☐3 Most of the time ☐4 Always

6-19.Have you ever disclose your HIV positive status to your spouse/steady partner?

☐1 Yes ☐2 No

6-20.In the past 6 months, have you had sex with someone other than your spouse/steady partner?

☐1 Yes, ☐☐ *persons* ☐2 No ***(Skip to Ques.6-22)***

6-21.How frequently did you use condoms when you had sex with him/her?

☐1 Never ☐2 Sometimes

☐3 Most of the time ☐4 Always

6-22.Can you get condoms when you need it?

☐1 Never ☐2 Sometimes

☐3 Most of the time ☐4 Always

CATS : Community Access to Treatment, Care and Support Study
National PLHIV Networks and Asia Pacific Network of People Living with HIV/AIDS (APN+)

SECTION 7 — ACCESS TO REPRODUCTIVE HEALTH AND FAMILY PLANNING SERVICE SHEET 1

7-1. Do you have child/children?

☐1 Yes ☐2 No ***(Skip to Ques.7-3)***

7-2. If yes, are any of these children known to be HIV positive?

☐1 Yes, number of children ☐☐ ☐2 No

7-3. Since being diagnosed as HIV positive, have you ever received counseling about child bearing?

☐1 Yes ☐2 No

☐3 I do not have a regular partner ***(Skip to Section 8)***

7-4. Do you now or did you since you were diagnosed of HIV have/had desire to have children?

☐1 Yes ☐2 No ***(Skip to Ques.7-8)***

7-5. Has a health care professional ever advised you not to have a child since you were diagnosed as HIV-positive?

☐1 Yes ☐2 No ☐3 Not applicable

7-6. Has a health care professional ever coerced you into being sterilized (a permanent form of providing contraception using surgical techniques) since you were diagnosed as HIV-positive?

☐1 Yes ☐2 No ☐3 Not applicable

7-7. In the last 12 months, have you or your spouse been coerced by a health care professional in relation to any of the following because of your HIV status?

7-7.1 Termination of pregnancy	☐1 Yes	☐2 No	☐3 Not applicable
7-7.2 Method of giving birth	☐1 Yes	☐2 No	☐3 Not applicable
7-7.3 Infant feeding practice	☐1 Yes	☐2 No	☐3 Not applicable

7-8. Did you/your spouse get pregnancy after getting HIV diagnosis?

☐1 Yes, ☐☐ *times* ☐2 No ***(Skip to Ques.7-12)*** ☐3 Not applicable ***(Skip to Ques.7-12)***

7-9. Was the pregnancy intended? (Please refer to your last pregnancy or present pregnancy if you were pregnant more than one time)

☐1 Yes ☐2 No ☐3 Not applicable

7-10.Did you/your spouse receive ART to prevent mother-to-child transmission of HIV during that pregnancy?

☐1 Yes ☐2 No ☐3 Not applicable

7-11.What was the outcome of that pregnancy

☐1 Terminated ☐2 Still pregnant

☐3 Gave birth at health facility ☐4 Gave birth at home

CATS : **Community Access to Treatment, Care and Support Study**
National PLHIV Networks and Asia Pacific Network of People Living with HIV/AIDS (APN+)

SECTION 7 — ACCESS TO REPRODUCTIVE HEALTH AND FAMILY PLANNING SERVICE SHEET 2

7-12. Currently, are you or your spouse taking/using any contraception? (Contraception means condoms, use of injectable or oral pills to prevent pregnancy)

☐1 Yes, name of the contraception ☐

☐2 No ☐3 Not applicable

7-13. Do you have access to contraception when you are in or need of it?

☐1 Yes ☐2 No ☐3 Not sure

7-14. Can you please tell me the name of the place where you can get contraception?

☐

7-15. How long does it take to reach to the nearest PMTCT site from your residence?

☐1 ☐☐☐ *mins* ☐2 Don't know ***(Skip to Section 8)***

7-16. How much do you need to spend to visit to the nearest PMTCT site from your residence? (Round trip transportation cost)

Specify amount in local currency ☐☐☐,☐☐☐,☐☐☐

CATS : Community Access to Treatment, Care and Support Study
National PLHIV Networks and Asia Pacific Network of People Living with HIV/AIDS (APN+)

SECTION 8 — HEPATITIS C VIRUS INFECTION

8-1. When you were diagnosed with HIV, were you ever offered information on Hepatitis C Virus (HCV) infection by your counsellor or doctor or nurse?

- ☐1 Yes
- ☐2 No

8-2. In subsequent health care visits, were you ever offered a hepatitis C test?

- ☐1 Yes
- ☐2 No ***(Skip to Section 9)***
- ☐3 I was diagnosed prior to HIV diagnosis

8-3. How were you tested for Hepatitis C Virus?

- ☐1 Self initiation
- ☐2 Doctor suggested it
- ☐3 I have not yet received the test
- ☐4 Others, specify ________

8-4. Did you have get the confirmatory blood test for Hepatitis C Virus?

- ☐1 Yes
- ☐2 No ***(Skip to Ques. 8-6)***

8-5. What was the test result?

- ☐1 Positive, HCV type ________
- ☐2 Negative ***(Skip to Section 9)***
- ☐3 Don't know ***(Skip to Section 9)***

8-6. Did you ever or currently receive the medication for HCV?

- ☐1 Yes, I already completed the medications
- ☐2 Yes, I am still on medication
- ☐3 No ***(Skip to Section 9)***

8-7. How much do/did you spend every month for your HCV medication?

- ☐1 local currency ☐☐☐,☐☐☐,☐☐☐
- ☐2 I am getting it/got it for free, from ________

**CATS :**	**Community Access to Treatment, Care and Support Study**<br>**National PLHIV Networks and Asia Pacific Network of People Living with HIV/AIDS (APN+)**
**SECTION 9**	**TUBERCULOSIS**

9-1. Were you ever diagnosed of having TB (detected of TB disease), after you were HIV positive?

☐1 Yes ☐2 No ***(Skip to Ques. 9-4)***

9-2. Did you receive the treatment for TB?

☐1 Yes ☐2 No ***(Skip to Ques. 9-4)***

9-3. Did you complete the treatment?

☐1 Yes ☐2 No

☐3 I don't know

9-4. Think about the past 6 months, during this period, did your doctor ask you if you had cough or night sweating or fever for over 3 weeks?

☐1 Yes ☐2 No

9-5. Think about the past 6 months, during this period, did your doctor ask you if you had recent contact with another person with TB?

☐1 Yes ☐2 No

9-6. How long does it take to reach to the nearest TB clinic from your residence?

☐1 ☐☐☐ *mins* ☐2 I don't know where TB clinic is ***(Skip to Section 10)***

9-7. How much do you need to spend to visit to the nearest TB clinic from your residence? (Average cost of round trip transportation cost)

local currency ☐☐☐,☐☐☐,☐☐☐

CATS :	Community Access to Treatment, Care and Support Study<br>National PLHIV Networks and Asia Pacific Network of People Living with HIV/AIDS (APN+)
**SECTION 10**	**HIV TREATMENT LITERACY**

I am going to read several statements about HIV treatment related issues. Please answer whether you think the statement is true or false. You can tell "don't know" if you are not sure about the statement.

	True	False	Don't Know
10-1. Once the HIV viral load results are "undetectable". ARVs should be stopped.	☐1	☐2	☐3
10-2. If ARVs are not taken at the right time(s) of day, HIV drug resistance can occur.	☐1	☐2	☐3
10-3. HIV is cured when the HIV viral load blood test result is "undetectable".	☐1	☐2	☐3
10-4. Condoms during sex are not needed when the HIV viral load blood test results are at "undetectable" levels.	☐1	☐2	☐3
10-5. It is better to take a half dose of HIV medications than to stop the HIV combination medications completely.	☐1	☐2	☐3
10-6. By engaging in risky sexual behaviours, one has a chance of getting infected with a drug resistance type of HIV	☐1	☐2	☐3
10-7. ARVs can cause unpleasant side effects (e.g. nausea, diarrhoea, vomiting).	☐1	☐2	☐3
10-8. Condoms are not needed if both sexual partners are HIV-positive.	☐1	☐2	☐3
10-9. If a person is experiencing side-effects such as diarrhoea, vomiting and /or nausea s/he should stop taking ARVs immediately.	☐1	☐2	☐3
10-10.Some illicit drugs can potentially compromise the effectiveness of HIV medications.	☐1	☐2	☐3
10-11.Providing ARVs to a HIV positive pregnant woman reduces the baby's risk of being infected with HIV.	☐1	☐2	☐3
10-12.There currently exists an HIV vaccine that prevents HIV infection.	☐1	☐2	☐3
110-3.ARVs can be taken at a different time of day on weekends or holidays.	☐1	☐2	☐3
10-14.All children born to HIV positive women who did not take PMTCT drugs are HIV-positive.	☐1	☐2	☐3
10-15.ARVs can be stopped as soon as you feel better.	☐1	☐2	☐3
10-16.Missing a few doses of ARV medicines can increase the amount of HIV virus in the body	☐1	☐2	☐3
10-17.Once a person starts to feel better, it becomes less important to take ARVs at the right time(s) of day.	☐1	☐2	☐3
10-18.ARVs help the body's immune system get stronger (CD4 increase).	☐1	☐2	☐3
10-19.The risk of opportunistic infections is reduced when CD4 level increases.	☐1	☐2	☐3
10-20.Physical exercise (e.g. yoga, tai chi) can help reduce stress levels among HIV patients.	☐1	☐2	☐3
10-21.HIV can be cured by taking ARVs.	☐1	☐2	☐3
10-22.ARV side-effects (such as liver problem, kidney problem, etc.) should be monitored through regular blood test.	☐1	☐2	☐3
10-23.CD4 count test measures how much HIV is in our body.	☐1	☐2	☐3
10-24.ARVs can be replaced by traditional herbs or herbal medicines.	☐1	☐2	☐3
10-25.As of now, ARVs need to be taken for life-long.	☐1	☐2	☐3

CATS : Community Access to Treatment, Care and Support Study
National PLHIV Networks and Asia Pacific Network of People Living with HIV/AIDS (APN+)

SECTION 11 — ACCESS TO INFORMATION TECHNOLOGY

11-1. Do you use a cell/mobile phone?

☐1 Yes ☐2 No ***(Skip to Ques. 11-4)***

11-2. Do you send/receive the text message?

☐1 Yes ☐2 No

11-3. How many times did you change your cell/mobile phone during the past one year? ☐☐ *times*

11-4. Do you have email account?

☐1 Yes ☐2 No ***(Skip to Ques. 11-6)***

11-5. During the past week, how many times did you check your personal email? ☐☐ *times*

11-6. Think about past seven days, how many hours did you work/spend in the internet? (This is to ask total number of hours spend in internet each day for past seven days) ☐☐ *hours*

11-7. Have you ever used the internet to find the HIV-related information?

☐1 Yes ☐2 No ***(Skip to Section 12)***

11-8. Were you satisfied with the information you found on internet?

☐1 Not at all ☐2 A little

☐3 Satisfied ☐4 Very much satisfied

CATS : Community Access to Treatment, Care and Support Study
National PLHIV Networks and Asia Pacific Network of People Living with HIV/AIDS (APN+)

SECTION 12 — ART INITIATION, REGIMEN AND ADHERENCE SHEET 1

12-1. Are you taking Anti Retroviral Therapy (ART) / HIV medicines now?

☐1 Yes ☐2 No

☐3 I don't know what is ART

(Please check again, if it is an understanding issue, if the respondent still does not know what is ART, Stop the interview)

12-2. When were you eligible to start ART? (When was the time your doctor told you that you should start ART?)

☐1 It was ☐☐ *years* ☐☐ *months* ☐☐ *days ago*

☐2 I am not eligible yet ***(Stop the interview)***

12-3. When did you start ART?

It was ☐☐ *years* ☐☐ *months* ☐☐ *days ago*

12-4. What is the name of the hospital/clinic you visited to start the ART?

[ ]

12-5. Are you still going to the same center?

☐1 Yes

☐2 No, I take ART from [ ] site

12-6. How long does it take to reach your current ART clinic from your residence?

☐1 Bus ☐2 Car

☐3 Walking ☐4 Bicycle

☐5 Others, specify [ ]

About ☐☐ *hours* ☐☐ *mins*

12-7. How much does it cost for you to visit the ART center? (Round trip transportation cost)

local currency ☐☐☐,☐☐☐,☐☐☐

12-8. Was your ARV medication regime ever changed?

☐1 Yes, ☐☐ *times* ☐2 No ***(Skip to Ques.12-10)***

12-9. What was the reason given to you for changing the regime?

☐1 Because of the side-effects ☐2 Because the ARV medicine was not available

☐3 Others, specify [ ]

12-10. How many times do you have to take your ART in a day? ☐☐ *times*

CATS :	Community Access to Treatment, Care and Support Study<br>National PLHIV Networks and Asia Pacific Network of People Living with HIV/AIDS (APN+)
**SECTION 12**	**ART INITIATION, REGIMEN AND ADHERENCE SHEET 2**

12-11. What are the names of the ART medicines you are taking now?

(If the respondents are not aware of the ARVs that they are taking, at the time of the interview, please follow the following steps:

☐ 1 Check if the respondent is carring the ARVs with him/her at the time of the interview. Take note the names. *(tick here if you had to do so)*

☐ 2 Check if the respondent is not carring the ARVs, take the pillbox photos from your bag, show it to the respondent and ask them to identify the ARVs. *(tick here if you had to do so)*

If the respondent is still unable to identify the ARVs that s/he is taking, Tick the choice 11 and move to the next question

- ☐ 1 Stavudine + Lamuvudin + Nevirapine
- ☐ 2 Stavudine + Lamuvudin + Efavirenz
- ☐ 3 Ziudovudine + Lamuvudin + Nevirapine
- ☐ 4 Ziudovudine + Lamuvudin + Efavirenz
- ☐ 5 Stavudine + Lamuvudin + Lopinavir/Ritonavir
- ☐ 6 Ziudovudine + Lamuvudin + Lopinavir/Ritonavir
- ☐ 7 Lamuvudin + Tenofovir + Nevirapine
- ☐ 8 Lamuvudin + Tenofovir + Efavirenz
- ☐ 9 Lopinavir + Ritonavir + Tenofovir
- ☐ 10 Others, specify ____________
- ☐ 11 I don't know the name of ARV medicines I am taking

12-12. Okay, let's think about 4 days back from today, please try to remember and tell me how many ARV medicine pills did you take in the morning/afternoon/night on each day? Also, tell what time did you take it.

	Medicine	Morning	Afternoon	Evening
**(a) Yesterday**		# of pills ☐☐<br>Time ☐☐ : ☐☐	# of pills ☐☐<br>Time ☐☐ : ☐☐	# of pills ☐☐<br>Time ☐☐ : ☐☐
**(b) 2 days ago**		# of pills ☐☐<br>Time ☐☐ : ☐☐	# of pills ☐☐<br>Time ☐☐ : ☐☐	# of pills ☐☐<br>Time ☐☐ : ☐☐
**(c) 3 days ago**		# of pills ☐☐<br>Time ☐☐ : ☐☐	# of pills ☐☐<br>Time ☐☐ : ☐☐	# of pills ☐☐<br>Time ☐☐ : ☐☐
**(d) 4 days ago**		# of pills ☐☐<br>Time ☐☐ : ☐☐	# of pills ☐☐<br>Time ☐☐ : ☐☐	# of pills ☐☐<br>Time ☐☐ : ☐☐

CATS : **Community Access to Treatment, Care and Support Study**
National PLHIV Networks and Asia Pacific Network of People Living with HIV/AIDS (APN+)

SECTION 12 — ART INITIATION, REGIMEN AND ADHERENCE SHEET 3

12-13. Did you miss taking any of your ARV medications, when was the last time you missed a dose?

☐1 Yes, it was ☐☐ *years* ☐☐ *months* ☐☐ *days ago*

☐2 No, I have never missed a dose ☐3 I don't remember, it was long time ago

12-14. We would like to get your best guess about how much of your ARV medications you have managed to take during the last month. Approximately what proportion (choose one from 0 to 100) of ARV medicines did you take during the last month?

*(__**Interviewer**__: Please make a mark on the line to show how many of the ARV pills the respondent managed to take in the last month (e.g. 0% means the respondent has not taken any medication, 50% means s/he has taken half the medication, 100% means s/he has taken every single dose)*

☐☐☐ %

0% 10% 20% 30% 40% 50% 60% 70% 80% 90% 100%

12-15. Have you ever missed an appointment with the ART clinic health care provider/doctor/nurse in the past 3 months?

☐1 Yes ☐2 No ***(Skip to Section 13)***

12-16. What are the reasons that you missed appointments with the health care providers in the last 3 months (Multiple choices)

12-16.1 Was busy with other things	☐1 Yes	☐2 No
12-16.2 Was out of town	☐1 Yes	☐2 No
12-16.3 Due to bad weather	☐1 Yes	☐2 No
12-16.4 Afraid of being arrested	☐1 Yes	☐2 No

12-16.5 Others, specify ________________

CATS :	Community Access to Treatment, Care and Support Study<br>National PLHIV Networks and Asia Pacific Network of People Living with HIV/AIDS (APN+)

SECTION 13	SIDE-EFFECTS OF ART

The following questions ask about symptoms you might have had during the past four weeks. Please tell the most appropriate response that describes how much you have been bothered by each the following symptoms.

	YES	NO	DON'T KNOW
**Short Term Side-Effects**			
13-1. Nausea and vomiting	☐1	☐2	☐3
13-2. Rash	☐1	☐2	☐3
13-3. Muscle aches and pains	☐1	☐2	☐3
13-4. Diarrhoea	☐1	☐2	☐3
13-5. Fatigue	☐1	☐2	☐3
13-6. Gas and bloating	☐1	☐2	☐3
13-7. Hair loss	☐1	☐2	☐3
13-8. Headache	☐1	☐2	☐3
13-9. Sleeping difficulties	☐1	☐2	☐3
13-10.Mood changes	☐1	☐2	☐3
**Long Term Side-Effects**			
13-11.Have you received the diagnosis of diabetes by your doctor after ART initiation?	☐1	☐2	☐3
13-12.Have you received the diagnosis of any heart-related diseases by your doctor after ART initiation?	☐1	☐2	☐3
13-13.Were you told by your doctor that the ART has caused body fat changes in your body?	☐1	☐2	☐3
13-14.Have you experienced the numbness in the limbs?	☐1	☐2	☐3
13-15.Were you told by your doctor that the ART has caused any problem in your kidney?	☐1	☐2	☐3
13-16.Were you told by your doctor that the ART has caused any problem in your liver?	☐1	☐2	☐3
13-17.Were you told by your doctor that the ART has caused any problem in your bone? (Osteopenia and osteoporosis)	☐1	☐2	☐3
13-18.Have you ever experienced jaundice after ART initiation?	☐1	☐2	☐3

CATS : Community Access to Treatment, Care and Support Study
National PLHIV Networks and Asia Pacific Network of People Living with HIV/AIDS (APN+)

SECTION 14 RELATIONSHIP WITH HEALTH CARE PROVIDERS

This section is designed to understand respondent's feelings on each of the statements on relationship with health care providers (include doctors, nurses, and technicians). Please request the respondent to rate answers in the 5 choices. Please read the 5 choices loudly. Request respondents to listen to the statements and think carefully on how strongly they agree or disagree with the statements

	Strongly disagree (1)	Disagree (2)	Neutral (3)	Agree (4)	Strongly agree (5)
14-1. HCP told me what the possible side effects of each of my ARVs are	☐1	☐2	☐3	☐4	☐5
14-2. HCP told me the changes to expect in my health when taking ARVs	☐1	☐2	☐3	☐4	☐5
14-3. It is difficult to ask the HCP about something I don't understand	☐1	☐2	☐3	☐4	☐5
14-4. The HCP make me feel comfortable when discussing any issue (including personal issue)	☐1	☐2	☐3	☐4	☐5
14-5. I'm sometimes insulted when talking to the HCP	☐1	☐2	☐3	☐4	☐5

Before closing, thank the respondent for giving time and providing valuable information.
Please re-aasure the respondent that the information will be treated confidentially. Ask the respondent if he or she has any question. Answer or refer them to correct information source, if necessary.

Ask the respondent if he or she is willing to be contacted for a follow-up interview in 9 months time (from now) and for subsequent follow-up in 2014 and 2015. If the respondent agrees, take note of contact details and wrap-up the session.

Interview end time ☐☐.☐☐
hh *mm*

Data collector's signature ☐

This section should be filled by the supervisor

Supervisor's name ________ Signature ☐

Review date ☐☐ ☐☐ 2 0 1 ☐
dd *mm* *yyyy*

End of the process!
